# Updating Research on Extracellular Vesicles of the Male Reproductive Tract in Farm Animals: A Systematic Review

**DOI:** 10.3390/ani14213135

**Published:** 2024-10-31

**Authors:** Pablo Martínez-Díaz, Ana Parra, Marina Montesdeoca, Isabel Barranco, Jordi Roca

**Affiliations:** Department of Medicine and Animal Surgery, Faculty of Veterinary Science, University of Murcia, 30100 Murcia, Spain; pablo.martinezd@um.es (P.M.-D.); ana.parra@um.es (A.P.); m.montesdeocagarcia@um.es (M.M.); isabel.barranco@um.es (I.B.)

**Keywords:** epididymal fluid, ectosomes, exosomes, extracellular vesicles, livestock species, microvesicles, seminal plasma

## Abstract

This systematic review examined research studies on extracellular vesicles (EVs) of the male reproductive tract in livestock species to summarize the research topics and methodologies used, key findings, and future directions. PubMed and Scopus were searched for time ranges up to 1 September 2024, and 1383 articles were identified. The application of screening and eligibility criteria resulted in the selection of 79 articles focusing on male reproductive EVs in livestock. Porcine and bovine male reproductive EVs were the most studied. A variety of EV isolation techniques were used, with ultracentrifugation being the most common. Characterization of male reproductive EVs in livestock was a weak point, with only 24.05% of the articles characterizing EVs according to MISEV guidelines. Inadequate characterization of EVs compromises the reliability of results. The results of 19 articles that provided a good characterization of EVs showed that male reproductive EVs from livestock species are phenotypically and compositionally heterogeneous. These papers also showed that these EVs would be involved in the regulation of sperm functionality. Research on male reproductive EVs in livestock species remains scarce, and further research is needed, which should include appropriate characterization of EVs and aim to find efficient methods to isolate them and assess their involvement in the functionality of spermatozoa and the cells of the female genital tract.

## Simple Summary

Over the past few decades, the scientific community has focused its attention on extracellular vesicles present in the male reproductive fluids of livestock species. Given the rapid increase in the number of articles focusing on this field of research, we conducted a systematic review to provide a summary of the aspects that have been investigated and the methodologies used to that end. Our findings indicate that extracellular vesicles from cattle and pigs have been the subject of the most extensive research to date. This research has employed a diverse range of isolation and characterization techniques. However, the characterization of extracellular vesicle samples remains a significant challenge, which undermines the rigor of the scientific evidence. The articles in which samples were properly characterized highlight the heterogeneity of extracellular vesicle composition and phenotype, as well as their involvement in regulating sperm physiology.

## 1. Introduction

Extracellular vesicles (EVs) are cell-derived nano-sized particles surrounded by lipid bilayers that carry active biomolecules, including proteins, lipids, and nucleic acids [1,2]. Extracellular vesicles are released by many of the body’s functional cells and play a key role in cell-to-cell communication by delivering molecular cargoes from donor to recipient cells, modulating their functional activity [2]. Extracellular vesicles have been isolated from all body fluids. With respect to reproductive-tract EVs, they have been isolated from virtually all male and female reproductive fluids, including epididymal [3], ejaculate [4], vaginal [5,6], uterine [7,8], oviductal [9,10], and follicular [11,12] fluids. Recent research would support the involvement of reproductive EVs in modulating key reproductive processes such as gamete maturation, fertilization, and embryo development and implantation [13].

Epididymal fluid (EF) and seminal plasma (SP) are the major male reproductive fluids. Epididymal fluid interacts with immature sperm as they travel through the epididymis and is essential for them to reach full maturity [14,15]. Seminal plasma, a mixture of the secretions mainly from the accessory sex glands, surrounds the sperm during and after ejaculation and is essential for the sperm to acquire full functional capacity, travel safely through the female genital tract, and successfully fertilize the oocyte [16]. Both male reproductive fluids are rich in functionally active molecules and EVs [17,18]. Indeed, SP contains more EVs than other major body fluids such as blood plasma and cerebrospinal fluid [19].

The membrane of the EV protects the molecular cargo from natural inactivators that circulate freely in body fluids, including proteases and RNases [4,20]. This protection allows the EV molecules to reach the recipient cells in an active form. Thus, the molecules carried by the EVs of male reproductive tract, rather than those circulating freely in the EF and SP, would be responsible for the functional activity attributed to the EF and SP, particularly in sperm maturation, regulation of the functional activity of mature spermatozoa, and immune tolerance of the female reproductive organs relative to sperm and embryos [4,21,22]. However, research on male reproductive EVs remains limited [23] and many results are inconsistent and open to speculation [4,24]. Research on male reproductive EVs has focused primarily on humans and less on other species of concern, such as livestock, whose productivity and sustainability depend on reproductive efficiency [25,26]. Improving the efficiency of reproductive management to produce more and better-quality meat from fewer animals and thereby reducing the environmental impact of livestock production is imperative as global meat consumption increases [27,28]. Therefore, the purpose of this systematic review was to summarize the current research on EVs from male reproductive fluids of livestock species. Specifically, the study aimed to (1) evaluate the quality and methodology of the research conducted, (2) synthesize the main results obtained, and (3) identify future research opportunities.

## 2. Methods

**Figure 1:**
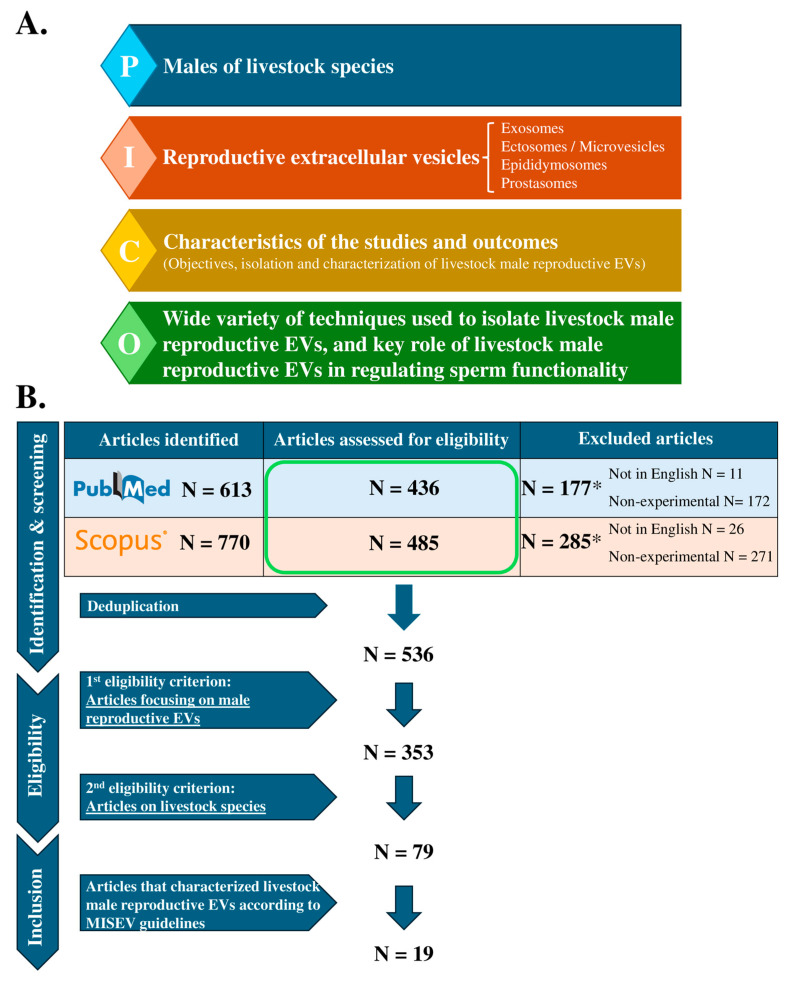
Flowchart illustrating the process of article selection: (**A**) the PICO (population, intervention, comparison, and outcome) principle and (**B**) Preferred Reporting Items for Systematic Reviews and Meta-Analyses (PRISMA) 2020 guidelines. N indicates the number of articles. * Some of the excluded papers met both exclusion criteria, i.e., they were not written in English and did not present experimental studies.

This systematic review was conducted according to the guidelines reported in the Preferred Reporting Items for Systematic Reviews and Meta-Analyses (PRISMA 2020) and in accordance with the PICO (population, intervention, comparison, and outcome) framework (Figure 1A).

### 2.1. Selection of Published Studies

The literature search was conducted in two databases, namely, PubMed (https://pubmed.ncbi.nlm.nih.gov/) and Scopus (https://www.scopus.com/). Both databases were last accessed on 1 September 2024. The signatory reviewers, P.M.-D. and M.M., performed the search independently using the same search strategy. All disagreements were resolved with the participation of two other signatory reviewers, namely, A.P. and J.R. Search criteria included combinations of the following keywords: epididymal, epididymis, ejaculate, semen, seminal plasma, sperm, spermatozoa, extracellular vesicle, exosome, microvesicle, epididymosome, and prostasome. The syntactic guidelines of each search engine were followed, as shown in Supplementary File S1. The search results obtained were screened for eligible articles according to the procedure described below.

### 2.2. Screening and Eligibility

These procedural steps were performed independently by the undersigned reviewers P.M.-D. and M.M., and any discrepancies were resolved by discussion with the undersigned reviewers A.P. and J.R. The set of articles identified in each database was screened to select only those articles reporting experimental results and written in English. Consequently, the search results consisting of reviews, books, book chapters, conference abstracts, letters, commentaries, responses, errata, and editorials were excluded. Articles from the two databases were then combined, and duplicate articles were removed. Two eligibility criteria were used to select eligible articles. The first was that eligible articles should contain the words extracellular vesicles, exosome, microvesicle, epididymosome, or prostasome in the title, abstract, and/or keywords. The terms exosomes and ectosomes (also known as microvesicles) define two types of extracellular vesicles that differ in their biogenesis. Epididymosomes and prostasomes define extracellular vesicles that are secreted in the epididymis and prostate gland, respectively. The second eligibility criterion was that eligible articles should describe studies performed in livestock species. Only articles containing terms related to horses, cattle, small ruminants (sheep and goats), pigs, chickens, or rabbits in the title, abstract, keywords and/or materials and methods section were selected.

### 2.3. Data Extraction

The undersigned reviewers P.M.-D. and M.M. independently extracted information from each eligible article, and any disagreements were discussed with the undersigned reviewers A.P., I.B, and J.R. Information was extracted in two separate steps. First, the title, reference information (i.e., authors, affiliations, country, journal, and year of publication), species, and keywords were recorded during the eligibility process to select the articles that focused on male reproductive EVs in livestock species. Second, information on the objectives and the methodology, including the procedures used to isolate and characterize EVs, as well as the main results, were recorded.

## 3. Results

### 3.1. Search Report

The systematic search of the databases yielded 613 articles in PubMed and 770 articles in Scopus, of which 436 and 485, respectively, were English-language articles reporting experimental results. After deduplication, 536 articles were assessed for eligibility criteria, resulting in 79 eligible articles focusing on male reproductive EVs in livestock species (Figure 1B). The most important items of the eligible articles are shown in Supplementary Table S1.

### 3.2. The Evolution of Publications over Time

**Figure 2:**
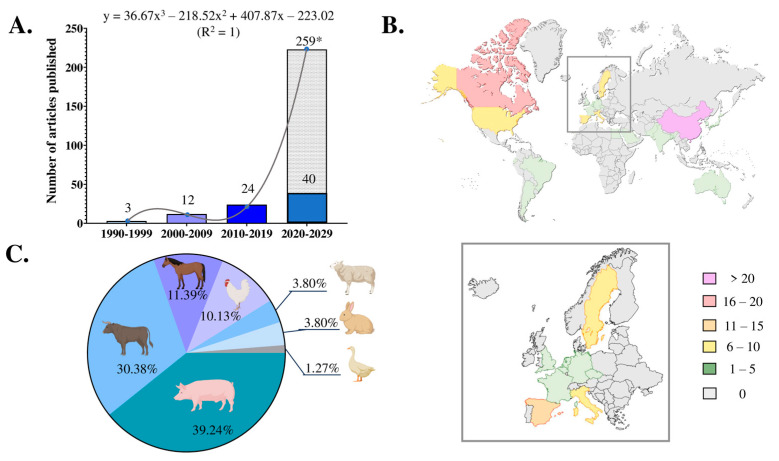
Research on extracellular vesicles in the male reproductive tracts of livestock species: (**A**) number of articles published per decade and trend, (**B**) articles published per country, and (**C**) articles published per species. * Number of articles that would be published by the end of the current decade.

The first article was published in 1998, and since then the number of articles published per decade has increased, with 2024 being the most productive year, with 11 articles published. In short, a total of three articles were published in the 1990s, twelve in the 2000s, twenty-four in the 2010s, and forty between 2020 and 1 September 2024. If the trend observed during the initial years of this decade were to persist, it is estimated that by the end of 2029, approximately 259 articles would be published within this decade. Taking this estimate into account, the publication trend over the decades would fit the formula y = 36.67x^3^ − 218.52x^2^ + 407.87x − 223.02 (R^2^ = 1) (Figure 2A).

### 3.3. Research Institutions and Publishing Journals

Research was conducted in laboratories from 23 different European, Asian, Oceanian, and American countries, with China being the most active (24 articles, 30.38%), closely followed by Canada (16 articles, 20.25%). Laboratories from Spain contributed twelve articles (15.19%), followed by Sweden and Italy (ten articles each, 12.66%) and the USA (seven articles, 8.86%) (Figure 2B). The most productive research institution was the University of Laval (Quebec, Canada), with 16 articles (20.25%). This was followed by the University of Murcia (Spain), with eight articles (10.13%); the South China Agricultural University, with six articles (7.59%); and the University of Uppsala (Sweden) and the University of Linköping (Sweden), with five articles each (6.33%). The articles were published in 44 different journals. *Biology of Reproduction* published 12 articles (15.19%); *Theriogenology*, 11 articles (13.92%); and *Frontiers in Veterinary Science* and *PLoS One*, four articles each (5.06%). The remaining 36 journals published three or fewer articles.

### 3.4. Livestock Species, Male Reproductive EV Origin, and Subset Differentiation

The research reported in the 79 eligible articles was mainly conducted on male reproductive EVs of pigs (31 articles, 39.24%) and cattle (24 articles, 30.38%); of the latter of which, two were conducted on buffalo bulls (2.53%). To a lesser extent, male reproductive EVs were also investigated in horses (9, 11.39%), chickens (8, 10.13%), sheep (3, 3.80%), rabbits (3, 3.80%), and ducks (1, 1.27%) (Figure 2C). The source of male reproductive EVs was variable, with seminal EVs (sEVs; isolated from SP) being the most studied (58 articles, 73.42%). Epididymal EVs were investigated in twenty-three articles (29.11%) and testicular EVs in one article (1.27%). Only one article investigated male reproductive EVs isolated from cell culture media (1.27%), specifically cells from the prostate, epididymis, and testis. A total of 26 articles (32.91%) reported the presence of male reproductive EV subpopulations. The subpopulations were isolated according to different criteria, including epididymal region (14, 17.72%), ejaculate fraction (3, 3.80%), secretory organ (1, 1.27%), sEV size (7, 8.86%), and sEV density (4, 5.06%).

### 3.5. Isolation Procedures

Approximately half of the studies used ultracentrifugation (UC) to isolate male reproductive EVs (41 articles, 51.90%). Of these, seven articles (8.86%) used density gradient UC. Precipitation was the isolation method used in five articles (6.33%), while size-exclusion chromatography (SEC) was the method used in two articles (2.53%). Many studies used a combination of two or more methods (29 articles, 36.71%). The most common combination was UC and SEC (17 articles, 21.52%). UC was also combined with precipitation (two articles, 2.53%), and SEC combined with ultrafiltration (eight articles, 10.13%). Other combinations were precipitation and immunocapture (one article, 1.27%) and ultrafiltration, precipitation, and immunocapture (one article, 1.27%). Two articles did not report the isolation of male reproductive EVs (2.53%).

### 3.6. Characterization of Male Reproductive EVs

**Figure 3:**
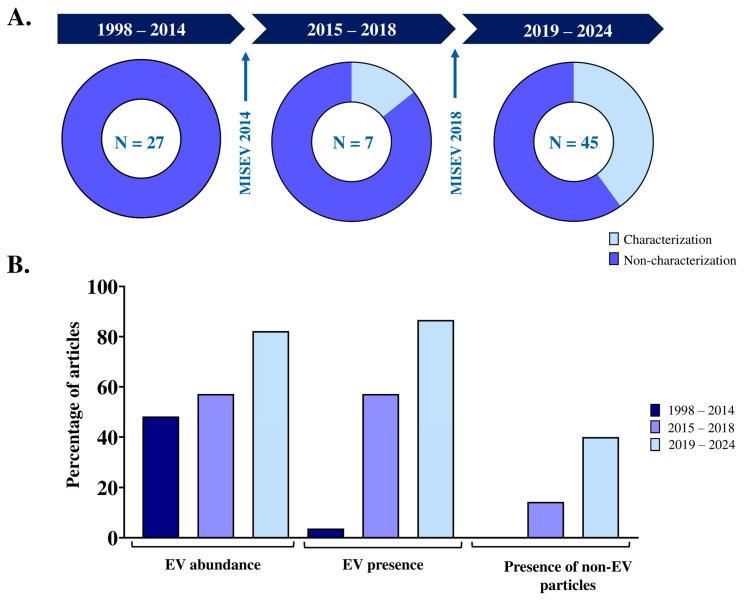
(**A**) Pie charts showing the proportion of research articles that characterized extracellular vesicles in the male reproductive tracts of livestock species according to the Minimal Information for Studies of Extracellular Vesicles (MISEV) guidelines, which recommend characterization of three attributes in EV samples: particle abundance, EV markers, and presence of non-EV particles. (**B**) Histograms showing which of the sEV attributes were characterized. Results are shown for three different time periods (1998–2014; 2015–2018; 2019–2024). These time periods were defined according to the publication of the MISEV guidelines (2014, 2018).

The International Society for Extracellular Vesicles (ISEV), through the Minimal Information for Studies of Extracellular Vesicles (MISEV) guidelines, recommends an orthogonal characterization of EV samples performed by analyzing three main attributes, namely, the abundance of nanoparticles, the identification of EVs, and the presence of non-vesicular extracellular particles (NVEPs) [29]. This recommendation was followed by only 19 of the 79 articles (24.05%). To date, three MISEV guidelines have been published, the first in 2014 [30], the second in 2018 [31], and the third in February 2024 [29]. According to this timing, three time periods were considered to analyze the evolution of the characterization of male reproductive EV samples in livestock, namely, from 1998 to 2014, from 2015 to 2018 and from 2019 to 2024. Only studies that evaluated the three main attributes recommended by MISEV were considered to have performed a satisfactory characterization of the male reproductive EV samples. As a result, none of the 27 articles published between 1998 and 2014 satisfactorily characterized the male reproductive EV samples. One (14.29%) of the 7 articles published between 2015 and 2018 satisfactorily characterized the male reproductive EV samples, and 18 articles of the 45 articles (40%) published between 2019 and 2024 satisfactorily characterized the male reproductive EV samples (Figure 3A). The most and least analyzed attributes of the livestock male reproductive EV samples and how the analysis of each attribute has improved over time are shown in Figure 3B. The least analyzed attribute is the presence of NVEPs.

Regarding the techniques used to characterize livestock male reproductive EVs, transmission electron microscopy (TEM) was the most frequently used (46 articles, 58.23%), followed by Western blot (35 articles, 44.30%), nanoparticle tracking analysis (NTA) (34 articles, 43.04%), and quantification of total protein concentration (25 articles, 31.65%). Other techniques also used were dynamic light scattering (DLS) (12 articles, 15.19%), flow cytometry (10 articles, 12.66%), scanning electron microscopy (SEM) (5 articles, 6.33%), and cryogenic electron microscopy (3 articles, 3.80%), atomic force microscopy, and quasi-elastic light scattering (2 articles each, 2.53%).

### 3.7. Research Topics

Articles were grouped into two main categories according to the research topic addressed, namely, male reproductive EV characterization and male reproductive EV functionality. Articles dealing with research on the phenotypic characteristics of livestock male reproductive EVs (i.e., size, morphology, EV markers, biochemical composition, etc.) were considered to be characterization research articles. Research articles aimed at deciphering the effects of livestock male reproductive EVs on target cells, such as spermatozoa or the functional cells of the female genital tract, as well as their functional effects on other physiological or pathological processes, were considered to be functional research articles. Articles that did not fit into either of the above two categories were included in the “other” category. Thus, 36 articles (45.57%) dealt with characterization topics, and 50 articles (63.29%) with functionality topics. Two articles (2.53%) were included in the “other” category because they dealt with the topic of the biogenesis of male reproductive EVs.

**Figure 4:**
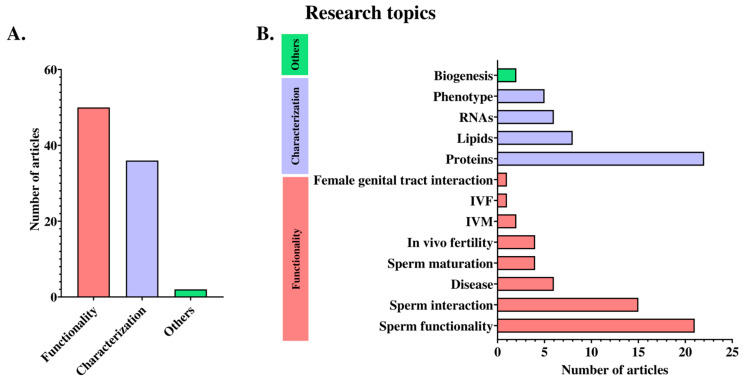
(**A**) Distribution of research articles among three categories according to whether they addressed research related to characterization, functionality, or other topics relating to extracellular vesicles in the male reproductive tracts of livestock species. (**B**) shows the specific research topic of the articles included in each of the three categories above. IVM: in vitro oocyte maturation; IVF: in vitro fertilization.

The characterization category included articles characterizing protein (22/79, 27.85%), lipid (8/79, 10.13%), and RNA (6/79, 7.59%) content, as well as phenotypic characterization (4/79, 5.06%). Regarding the functionality category, articles investigated the interaction of livestock male reproductive EVs with spermatozoa (15/79, 18.99%) and with cells of the female genital tract (1/79, 1.27%), as well as the effect of livestock male reproductive EVs on sperm maturation (4/79, 5.06%) and sperm functionality (21/79, 26.58%). Other articles on male reproductive EV functionality evaluated the effects of livestock male reproductive EVs on in vitro oocyte maturation (2/79, 2.53%) and in vitro fertilization (1/79, 1.27%), or their roles in in vivo (in)fertility (4/79, 5.06%) and diseases (6/79, 7.59%) (Figure 4).

### 3.8. Main Results

Proper characterization of EVs according to the MISEV guidelines is necessary to ensure the quality, rigor, and reproducibility of EV experiments [32]. Therefore, only the results of the 19 articles (24.05%) that properly characterized male reproductive EV samples by analyzing nanoparticle abundance, identifying EVs, and quantifying the presence of NVEPs, were reviewed. These nineteen articles dealt with seminal plasma EVs (sEVs), including seven articles focusing on sEV characterization (Table 1) and twelve articles focusing on sEV functionality (Table 2).

#### 3.8.1. Results of the Characterization Studies Relating to sEVs

In order of publication, the first study was conducted by Skalnikova et al., who isolated and characterized EVs from three porcine body fluids, namely, blood plasma, cerebrospinal fluid, and SP. They found that SP was the most EV-rich body fluid, reaching a concentration of 10^10^ per milliliter [19]. The following two chronological articles unraveled the protein composition of chicken and porcine sEVs using omics [33,34]. They identified proteins in the cargo of sEVs that are associated with sperm maturation and functionality and the establishment of an appropriate immune environment in the female genital tract for optimal sperm and embryo survival. In addition, Barranco et al. found quantitative differences in functionally relevant proteins between large and small sEVs [34]. Another article examined the cargo of sEVs in transforming growth factor beta (TGF-β) isomers and showed that sEVs contain the three TGF-β isomers (1–3), mainly in the outer coronal layer surrounding the EV membrane [35]. Barranco et al. focused on the use of high-sensitivity flow cytometry for the characterization of porcine sEVs [36]. They demonstrated the existence of different subtypes of sEVs depending on their expression of CD9, CD63, and CD81, the tetraspanins recognized as EV protein markers. In addition, Barranco et al. found expression of the transmembrane protein CD44 in the entire sEV population [36]. Therefore, CD44 could be considered to be a universal protein marker for porcine sEVs. The lipid profile [37] and the structure and morphology [38] of different porcine sEV subsets have been the focus of the most recent articles. Martínez-Díaz et al. demonstrated the existence of qualitative and quantitative differences in the lipid composition between large and small sEVs [37]. Using cryogenic electron microscopy, Parra et al. demonstrated the high heterogeneity in structure and morphology of sEVs and suggested that large sEVs are mainly derived from seminal vesicles and small sEVs are mainly derived from the epididymis and prostate [38].

#### 3.8.2. Results of the Functional Studies on sEVs

Six of the twelve articles focusing on sEV functionality examined the role of sEVs in sperm functionality, and four of these studies focused on the role of sEVs in sperm motility. Ding et al. compared the proteome and transcriptome of sEVs from pigs with high and low sperm motility. They found proteins, microRNAs (miRNAs), long non-coding RNAs (lncRNAs), and messenger RNAs (mRNAs) that were quantitatively differentially expressed, many of which were directly involved in sperm motility [39]. Studies with a similar experimental design were performed by Ding et al., Zhao et al., and Zhang et al., all in pigs, comparing the composition of sEVs from boars with high and low sperm motility. Ding et al. found quantitative differences in circular RNAs and established the motility-promoting and apoptosis-inhibiting functions of sEV-associated circ-CREBBP [40]. Zhao et al. found quantitative differences in miRNAs targeting genes involved in transcriptional regulation of the RNA polymerase II promoter, regulation of gene expression, and intracellular signaling [41]. Finally, Zhang et al. found quantitative differences in proteins and metabolites, some of which are involved in purine metabolism [42]. Xu et al. investigated the functional interaction of sEVs with porcine spermatozoa. The results suggested that EZRIN, a CD44 linker, was involved in sEV–sperm interaction and that sEVs would be involved in inhibiting acrosomal reaction and thus sperm fertilization capacity [43]. The study conducted by Chen et al. was the only one to analyze the effect of sEVs on sperm preservation, and the results showed that they have an important effect. The addition of sEVs to the semen storage extender reduces the generation of reactive oxygen species (ROS) by the mitochondrial sheet of porcine spermatozoa, thus improving the sperm preservation capacity [44].

The influence of sEVs on in vivo and in vitro fertility outcomes was investigated in three studies. Sakr et al. reported quantitative differences in sEV miRNAs between male rabbits showing differences in in vivo fertility outcomes [45]. Mateo-Otero et al. investigated whether sEVs affect in vitro maturation of porcine oocytes. They found that sEVs were able to bind to cumulus cells but not to immature oocytes. Consequently, sEVs had no effect on oocyte nuclear maturation, but they did modulate gene expression in cumulus cells, especially large sEVs [46]. Barranco et al. evaluated whether sEVs affect in vitro fertility outcomes and reported that the addition of sEVs to the fertilization medium decreased sperm–zona pellucida binding, thereby reducing oocyte fertilization rates. This was because the sEVs altered sperm metabolism [47].

Finally, three of the twelve studies examined the involvement of sEVs in specific viral diseases of livestock species. Su et al. demonstrated that sEVs from reticuloendotheliosis-infected chickens contained genomic RNA and viral proteins and that sEVs were more efficient than free virions for virus transmission [48]. Similarly, for the avian leukosis virus subgroup J, Liao et al. showed that sEVs from infected chickens contain viral RNA and proteins. They also showed that these sEVs can infect healthy hens after artificial insemination and facilitate vertical transmission of the disease to offspring [49]. Finally, Carossino et al. showed in horses that sEV-associated eca-mir-128 is involved in the regulation of a chemokine axis involved in persistent equine arteritis virus infection [50].

## 4. Discussion

This systematic review provides an analysis of experimental studies conducted on EVs of the male reproductive tracts of livestock species, with the aim of summarizing the current state of research in this field. In particular, the review has made it possible to identify the technical standard of the studies carried out and to highlight the main results obtained. In addition, this systematic review has allowed the authors to identify the weaknesses of the existing studies and the research niches for future studies.

The first point to note is the relatively small number of published articles focusing on male reproductive EVs from livestock species. This was to be expected, considering that EVs from male reproductive fluids have been studied less than those from other body fluids such as blood plasma, serum, or urine [23]. In addition, a large fraction of the few existing studies on male reproductive EVs have been conducted in humans and laboratory animals [24]. The oldest eligible study was published only 26 years ago, clearly indicating that research on male reproductive EVs in farm animals is a young field of science. Since then, the number of published articles has followed an upward trend, indicating that livestock male reproductive EVs have attracted the interest of researchers. This interest is particularly evident among researchers from China, Canada, and Spain. The journal analysis showed that most articles were published in journals related to reproductive biology, such as *Biology of Reproduction* and *Theriogenology*. Surprisingly, there were no articles published in EV-specific journals, such as *Journal of Extracellular Vesicles*, *Journal of Extracellular Biology*, and *Journal of Extracellular Vesicles and Circulating Nucleic Acids*. This would suggest that issues of reproductive (dys)function, rather than male reproductive EVs, have been the target of published research. The male reproductive EVs that have been most studied are those from pigs and cattle. This is reasonable, as these are the two livestock species that contribute most to global wealth [51]. They are also the two species in which reproductive technologies, such as artificial insemination or in vitro production and transfer of embryo, are widely used [52,53,54].

Regarding the origin of male reproductive EVs, in most articles they were isolated from SP. Seminal plasma is a mixture of secretions from different reproductive organs, such as testes, epididymides, and, especially, accessory sex glands [16]. This would contribute to the claimed heterogeneity of the sEV population [36,55,56,57]. There is also a notable number of articles focusing on male reproductive EVs from specific sources, mainly from the epididymis, with research conducted to investigate the role of epididymal EVs in sperm maturation [15].

Looking at the technical methods used, the first consideration is EV isolation, as it defines the quantity, quality, and purity of EV samples [58]. In the published articles, a variety of methods were used. This indicates that there is still no ideal method to isolate male reproductive EVs. The most used method was UC [23], which is reasonable since it was the first method used to isolate EVs and is still considered the gold standard [59]. However, it may not be the best option for EV isolation because NVEPs co-sediment with EVs and are a major source of biological contamination [60,61]. In addition, UC promotes EV aggregation and negatively affects EV integrity, morphology, and functionality [62,63]. Perhaps these limitations of UC are the reason why alternative methods have also been used. Many studies have used a combination of two or more isolation methods, and most of them include SEC as one of the methods. The main reason for this choice may be that SEC allows the isolation of EVs which are more intact and less contaminated compared to those isolated with UC [58]. The utility of other methods used in combination with SEC would be to concentrate the initial sample prior to SEC, as SEC is more suitable for small sample volumes [64].

In addition to isolation, EV characterization is another key technical step in any research focused on EVs. It is essential to ensure that the sample is enriched in EVs to guarantee the robustness and reliability of the results obtained [31]. To properly characterize EV samples, ISEV, through MISEV guidelines, recommends determining the abundance of nanoparticles in the samples, verifying that these nanoparticles are predominantly EVs by detecting some EV-specific protein markers, and ensuring that NVEPs are present at low abundances [29,31]. Unfortunately, most studies did not consider this orthogonal approach to EV sample characterization, which is particularly surprising as to recent studies, as the first MISEV guidelines were published in 2014. Fortunately, the number of studies following the MISEV guidelines has increased over time. The most commonly used techniques to address this orthogonal characterization were NTA, total protein concentration, Western blot, and TEM. These are the techniques used in most studies of EVs, regardless of sample source [23,32].

In terms of research topics, there are articles that focus on the characterization of male reproductive EVs and the analysis of their functional roles, the latter being the majority. This distribution contrasts with that reported by Parra et al. in another recent systematic review on male reproductive EVs. They reported a preponderance of characterization studies. However, they included male reproductive EV studies in all species, including humans, laboratory animals, pets, and exotic species [24]. Looking at the characterization studies, most of them were performed in pig sEVs and highlight their heterogeneity, not only in terms of size and shape, but also as to the presence of EV-specific protein markers [36,55]. This heterogeneity may be due to the different cellular sources of sEVs, as discussed above. Three articles addressed the characterization of the sEV cargo, specifically the lipidome and the proteome, and highlighted the presence of proteins involved in sperm maturation and sperm functionality. This is a clear indication that the sEVs would be important players in the functional development of the spermatozoa. With only three articles characterizing the sEV cargo, more efforts are needed to characterize the sEV composition, as their functions depend on the cargo [2]. This includes the proteins, lipids, and nucleic acids that are transferred to recipient cells, whether spermatozoa or functional epithelial cells of the female reproductive tract [65].

Articles on sEV functionality have shown that sEVs from livestock species are involved in the regulation of important sperm functions such as motility [39,41,42,44], acrosome reaction [43], and antioxidant capacity [44]. The miR-222 would be an sEV cargo molecule particularly relevant to the role of sEVs in regulating sperm motility [39,41]. Regarding the acrosome reaction, sEVs would have an inhibitory effect on it and thus reduce the fertilization capacity of the sperm [43]. In this regard, in vitro studies conducted by Barranco et al. showed that sEVs alter sperm metabolism, thus reducing their fertilizing capacity [47]. No studies have addressed the role of sEVs in the functionality of cells of the female genital tract. Therefore, this should be a challenge for future research. In this regard, the study by Padilla et al. showed that sEVs carry TGF-βs, molecules that are involved in promoting maternal immune tolerance, thus facilitating early embryonic development and implantation [35,66].

Finally, it should be mentioned that this systematic review may have some methodological limitations that may have led to the exclusion of some articles. Although the PRISMA-based search strategy was designed to include as many articles as possible, it is reasonable to assume that this was not the case. For example, articles written in languages other than English or those that did not include the search terms in the title or abstract were not included. Another limitation could be the exclusion from the research review of articles that did not follow the MISEV guidelines for the characterization of EVs. In this regard, it is important to remember that proper characterization of EVs is essential to obtain robust, reliable, and reproducible results.

## 5. Conclusions

Many of the studies on male reproductive-tract EVs in livestock species to date lack adequate characterization of sEVs, undermining the robustness and reliability of their findings. This, combined with the variety of methods used to isolate livestock male reproductive EVs, makes it difficult to replicate experiments and compare results across studies.

The studies that have focused on characterizing sEVs have revealed their diversity in size, shape, and molecular composition. Studies investigating the functionality of sEVs have highlighted their key roles in some functional characteristics of spermatozoa, particularly motility. However, the lack of studies evaluating the effects of sEVs on the functional responses of cells of the female genital tract is noticeable. These gaps highlight the need for further studies investigating male reproductive EVs in livestock species. Future studies should prioritize the development of methods for efficient isolation of livestock male reproductive EVs and then evaluate their role in sperm and female reproductive-tract function. Efforts should be directed towards the rigorous and comprehensive characterization of isolated male reproductive EVs, which is essential to obtain robust and reproducible results.

## Figures and Tables

**Table 1:** The seven eligible and included research articles with results focused on seminal extracellular vesicle (sEV) characteristics.

Authors and Reference	Species	EV Isolation Procedure	EV Characterization Techniques	Research Topic	Main Conclusion
Skalnikova et al. [19]	Porcine	Serial centrifugations and UC	TEM, flow cytometry (CFSE), Western blot (TSG101, Alix, lamin A/C, β-tubulin, UQCRC1)	Characterization of EVs isolated from blood plasma, cerebrospinal fluid, and seminal plasma	Seminal plasma is richer in EVs than are cerebrospinal fluid and blood plasma
Luo et al. [33]	Chicken	Precipitation and UC	BCA protein assay, TEM, SEM, NTA, Western blot (TSG101, CD9, CD63, CD81, CD44, HSP70, calnexin)	Proteomic characterization of chicken sEVs	sEVs contain proteins related to sperm maturation and function
Barranco et al. [34]	Porcine	Serial centrifugations, ultrafiltration, and SEC	DLS, NTA, TEM, flow cytometry (CD63, HSP90β, albumin)	Proteomic characterization of two differently sized sEV subtypes	Small and large sEVs differ in proteins with biological functional relevance
Padilla et al. [35]	Porcine	Serial centrifugations, ultrafiltration, and SEC	Micro-BCA protein assay, NTA, DLS, cryo-EM, flow cytometry (CD63, CD44, HSP90β, albumin)	Presence of TGF-β isoforms in sEVs	sEVs carry TGF-β isoforms mainly in the outer coronal layer
Barranco et al. [36]	Porcine	Serial centrifugations, ultrafiltration, and SEC	BCA protein assay, DLS, TEM, Western blot (albumin), flow cytometry (CFSE, CD9, CD44, CD63, CD81, HSP90β, albumin)	Immunophenotypic characterization of differently sized sEV subsets from different ejaculate fractions	Highly sensitive flow cytometry allows the identification of sEV subsets
Martínez-Díaz et al. [37]	Porcine	Serial centrifugations, ultrafiltration, and SEC	Micro-BCA protein assay, NTA, DLS, TEM, flow cytometry (CD63, HSP90β, albumin)	Lipidomic characterization of two differently sized sEV subsets	Small and large sEVs show differences in the lipidomic profile
Parra et al. [38]	Porcine	Serial centrifugations, ultrafiltration, and SEC	Micro-BCA protein assay, cryo-EM, DLS, flow cytometry (CD63, HSP90β, albumin)	Phenotypical characterization of sEV subsets using cryo-EM	sEVs are structurally and morphologically heterogeneous

Abbreviations. Alix: ALG-2-interacting protein X; BCA: Bicinchoninic acid; CD44: Cluster of differentiation 44; CD63: Cluster of differentiation 63; CD81: Cluster of differentiation 81; CD9: Cluster of differentiation 9; CFSE: Carboxyfluorescein succinimidyl ester; Cryo-EM: Cryogenic electron microscopy; DLS: Dynamic light scattering; HSP70: Heat shock protein 70; HSP90β: Heat shock protein 90kDa beta; NTA: Nanoparticle tracking analysis; SEC: size-exclusion chromatography; SEM: Scanning electron microscopy; TEM: Transmission electron microscopy; TGF-β: Transforming growth factor beta; TSG101: Tumor susceptibility gene 101; UC: Ultracentrifugation; UQCRC1: Cytochrome b-c1 complex subunit 1.

**Table 2:** The twelve eligible and included research articles with results focusing on functional aspects of seminal extracellular vesicles (sEVs) in livestock.

Authors and Reference *	Species	EV Isolation Procedure	EV Characterization Techniques	Research Topic	Main Conclusions
Ding et al. [39]	Porcine	Centrifugation and UC	TEM, NTA, Western blot (Alix, CD63, TSG101, calnexin)	Role of sEV-associated RNAs and proteins in regulating sperm motility and apoptosis	sEV-miR-222 improves sperm motility and reduces sperm apoptosis
Ding et al. [40]	Porcine	Serial centrifugations and UC	TEM, NTA, Western blot (calnexin, Alix, TSG101, CD81, CD9)	Role of sEVs in sperm motility	circRNA profile differs between sEVs from boars with high or low sperm motility
Zhao et al. [41]	Porcine	Centrifugation, UC, and UC with gradients	TEM, NTA, Western blot (HSP70, TSG101, calnexin)	Relationship between miRNA profiles of sEVs and sperm motility	sEV miRNA expression differs between low and high motile sperm samples
Zhang et al. [42]	Porcine	Serial centrifugations and UC	NTA, TEM, Western blot (TSG101, Alix, CD9, calnexin)	Characterization of the proteomic and metabolomic profile of sEVs from boars with high and low sperm motility	sEVs carry several proteins and metabolites that modulate sperm motility
Xu et al. [43]	Porcine	Serial centrifugations, UC, and UC with gradients	TEM, NTA, Western blot (CD9, CD63, calnexin), BCA protein assay	Interaction between sEVs and sperm and effects on sperm functionality	sEVs impair acrosome reaction and IVF outcomes
Chen et al. [44]	Porcine	Serial centrifugations and UC	BCA protein assay, TEM, NTA, Western blot (HSP70, CD63, calnexin)	Role of sEVs in sperm preservation	sEVs inhibit lysophosphatidylcholine metabolism and reduce mitochondrial ROS production
Sakr et al. [45]	Rabbit	Serial centrifugations, SEC, and ultrafiltration	TEM, NTA, Western blot (HSP70, CD9, Alix, calnexin)	Characterization of the miRNA expression profile of sEVs from rabbit bucks with different fertility status	sEVs from fertile and subfertile rabbits differ in miRNAs related to spermatogenesis and sperm quality
Mateo-Otero et al. [46]	Porcine	Serial centrifugations, ultrafiltration, and SEC	BCA protein assay, NTA, DLS, TEM, flow cytometry (CFSE, CD44, HSP90β, albumin)	Role of sEVs on cumulus-oocyte complexes during IVM	sEVs bind to cumulus cells but not to oocytes. They do not affect oocyte maturation, but can modulate cumulus cell function
Barranco et al. [47]	Porcine	Serial centrifugations, ultrafiltration, and SEC	Micro BCA protein assay, cryo-EM, flow cytometry (CD81, HSP90β, albumin)	Effects of sEVs on IVF outcomes	sEVs decrease sperm–ZP binding and impair IVF process
Su et al. [48]	Chicken	Serial centrifugations, ultrafiltration, precipitation, and immunocapture (CD63)	Western blot (CD9, CD63, Alix, GRP94), TEM, BCA protein assay	Role of sEVs in reticuloendotheliosis virus (REV) transmission	sEVs from REV-positive chickens contain viral genomic RNA and viral proteins
Liao et al. [49]	Chicken	Serial centrifugations, precipitation, and immunocapture (CD63)	TEM, immunogold labeling (CD63), NTA, Western blot (CD81, CD63, TSG101, GRP78)	Role of sEVs in vertical transmission of avian leukosis subgroup J virus (ALV-J)	sEVs from subgroup ALV-J-infected roosters can transmit ALV-J infection to host cells
Carossino et al. [50]	Horse	Precipitation	TEM, immunogold labeling (CD9), Western blot (CD9, HSP70, calreticulin)	Role of sEV-associated miRNAs in persistent equine arteritis virus infection	sEV-associated eca-mir-128 modulates CXCL16 expression in the reproductive tract

Abbreviations. Alix: ALG-2-interacting protein X; BCA: Bicinchoninic acid; CD44: Cluster of differentiation 44; CD63: Cluster of differentiation 63; CD81: Cluster of differentiation 81; CD9: Cluster of differentiation 9; CFSE: Carboxyfluorescein succinimidyl ester; circRNA: circular RNA; Cryo-EM: Cryogenic electron microscopy; CXCL16: Chemokine ligand 16; DLS: Dynamic light scattering; GRP78: Glucose-regulated protein; HSP70: Heat shock protein 70; HSP90β: Heat shock protein 90kDa beta; IVF: In vitro fertilization; IVM: in vitro oocyte maturation; REV: reticuloendotheliosis virus; ROS: Reactive oxygen species; SEC: Size-exclusion chromatography; TEM: Transmission electron microscopy; UC: Ultracentrifugation; miRNA: MicroRNA; TSG101: Tumor susceptibility gene 101; ZP: Zona pellucida. * The reference is the same used in the text.

## Data Availability

Not applicable.

## Supplementary Materials

The following supporting information can be downloaded at: https://www.mdpi.com/article/10.3390/ani14213135/s1, File S1: Search strategy; Table S1: Eligible and included articles.
