# Supplementary material for: Updating Research on Extracellular Vesicles of the Male Reproductive Tract in Farm Animals: A Systematic Review
Source: Animals (Basel). 2024 Oct 31;14(21):3135. doi: 10.3390/ani14213135 (PMC11545059; doi:10.3390/ani14213135)
Supplement: Supplementary file 1 [file animals-14-03135-s001.zip › Supplementary file S1_Search strategy.docx]

**Search strategy**

PubMed

Terms to query:

(semen[Title/Abstract]) OR (seminal plasma[Title/Abstract]) OR (epididymis[Title/Abstract]) OR (ejaculate[Title/Abstract]) OR (sperm[Title/Abstract]) OR (spermatozoa[Title/Abstract])

AND

(exosome[Title/Abstract]) OR (extracellular vesicles[Title/Abstract]) OR (microvesicle[Title/Abstract]) OR (prostasome[Title/Abstract]) OR (epididymosome[Title/Abstract])

Scopus

Terms to query:

(TITLE-ABS (semen) OR TITLE-ABS (seminal AND plasma) OR TITLE-ABS (epididymis) OR TITLE-ABS (ejaculate) OR TITLE-ABS (sperm) OR TITLE-ABS (spermatozoa))

AND

(TITLE-ABS (exosome) OR TITLE-ABS (extracellular AND vesicle) OR TITLE-ABS (microvesicle) OR TITLE-ABS (epididymosome) OR TITLE-ABS (prostasome))
